# Deep Neural Network for Point Sets Based on Local Feature Integration

**DOI:** 10.3390/s22093209

**Published:** 2022-04-22

**Authors:** Hao Chu, Zhenquan He, Shangdong Liu, Chuanwen Liu, Jiyuan Yang, Fei Wang

**Affiliations:** 1 School of Robotics and Engineering, Northeastern University, Shenyang 110167, China; chuhao@mail.neu.edu.cn (H.C.); 2002006@stu.neu.edu.cn (S.L.); 2002005@stu.neu.edu.cn (C.L.); 2 Queen Mary School of Engineering, Northwestern Polytechnical University, Xi’an 710060, China; 3719@mail.nwpu.edu.cn

**Keywords:** deep learning, local feature integrating, object classification, point cloud, part segmentation

## Abstract

The research of object classification and part segmentation is a hot topic in computer vision, robotics, and virtual reality. With the emergence of depth cameras, point clouds have become easier to collect and increasingly important because of their simple and unified structures. Recently, a considerable number of studies have been carried out about deep learning on 3D point clouds. However, data captured directly by sensors from the real-world often encounters severe incomplete sampling problems. The classical network is able to learn deep point set features efficiently, but it is not robust enough when the method suffers from the lack of point clouds. In this work, a novel and general network was proposed, whose effect does not depend on a large amount of point cloud input data. The mutual learning of neighboring points and the fusion between high and low feature layers can better promote the integration of local features so that the network can be more robust. The specific experiments were conducted on the ScanNet and Modelnet40 datasets with 84.5% and 92.8% accuracy, respectively, which proved that our model is comparable or even better than most existing methods for classification and segmentation tasks, and has good local feature integration ability. Particularly, it can still maintain 87.4% accuracy when the number of input points is further reduced to 128. The model proposed has bridged the gap between classical networks and point cloud processing.

## 1. Introduction

Object classification and part segmentation are playing an increasingly important role in many fields. It is a hot topic to collect and process signals through sensors so that machines can perceive objects. In recent years, with the development of 3D data-capturing devices such as depth and LIDAR sensors, point clouds collection has become more and more convenient. Point cloud, a particularly important type of geometric point sets, is close to the original sensor data and can fully reflect the geometric information of the object. Compared with traditional image-based approaches, point cloud avoids the irregularities and complexity of grid, and thus it has a simple and unified structure that is easier to process. Meanwhile, the representation of raw point cloud can make full use of the geometric and topological structure of 3D space and bring better performance. For the characteristics mentioned above, the study and implementation of point clouds have diverse realistic applications in many fields, such as (Geographic Information System) GIS remote sensing [1], autonomous vehicles [2], hand pose estimation [3], underground mining environment [4], face recognition [5], city building reconstruction [6], multi-target recognition [7,8], and so on.

Until now, few studies have involved networks that process raw point cloud data directly. PointNet [9] has designed a deep net architecture suitable for unordered point sets, being regarded as a pioneering effort in this field. However, the success of convolutional architectures depends on the extraction of local features, which PointNet cannot do. PointNet++ [10] has resolved such deficiency, whereas it fails to take the mutual learning of neighboring points into consideration. The key of point cloud processing is to solve the problem of local feature integration.

In this work, we construct a simple and general 3D deep learning framework that directly takes point clouds as input and produces either class labels for the entire input or per point segment labels for each point of the input. The intuition behind the model is very straightforward: strengthening the network’s ability to integrate local features while improving the robustness of the system. Although the previous network can extract local features, it does not have strong robustness when the sampling points are reduced. Specifically, PointNet treats each point cloud as an isolated point for feature extraction, but ignores local feature aggregation. PointNet++ takes into account the extraction of point cloud features in the local area, but ignores the internal connection between points. If we express centroid points according to structural information extracted from the neighboring points and promote the further integration of feature information among points in the local region, it will definitely improve the ability to integrate local features and the performance of the whole network model. Meanwhile, the neighboring points of each centroid can learn from each other to improve the robustness of the network.

In this paper, the novel Modified Set Abstraction (MSA) module was adopted as the feature extraction backbone of the model, which consists of four layers: Sampling layer, Recombination layer, R3Block layer, and Maxpooling layer. The sampling layer is responsible for selecting a fixed number of point clouds as the centroids of the local regions, then the recombination layer finds the neighboring points of the centroids and performing further feature fusion. The R3Block (Residual-like block in 3D point cloud processing) layer is used for replacing the MLP-based mini PointNet layer to encode local features and the maxpooling layer reduces the feature dimensionality finally. In particular, the R3Block layer is a structure of the Residual-like block built on the ideas of the classical network, which adds the learning of neighboring points in 3D point cloud processing to improve the integration of local features. Extensive experiments show that our model is able to process raw point sets efficiently and robustly on the 3D object datasets and the indoor remote-sensing datasets.

In summary, the contributions of this work are three-fold:

- A novel point-cloud based framework, which utilizes the MSA module to improve the ability of local feature integration and the robustness of the network while bridging the gap between classical network and point cloud processing.
- The performance of proposed approach is evaluated on public datasets, from shape classification to scene semantic segmentation, which shows that the performance is significantly improved compared to most existing methods.
- The efficiency and stability of the model are proved by the robustness test and the intuitive visualization of segmentation results.

## 2. Related Works

Many traditional point cloud feature descriptors [11,12,13,14] are handcrafted for specific tasks, which would significantly restrict the algorithm generality. However, with the fast and tremendous progress of deep learning methods, many deep neural networks for point cloud processing have been proposed. These methods can be categorized into two groups: one is transforming point clouds into other 3D representations, and the other is consuming point clouds directly.

### 2.1. Transforming Point Clouds into Other 3D Representations

Previous approaches usually focus on transforming point clouds into other 3D representations, like collections of images [15,16,17] or 3D voxel grids [18,19], before the data were fed into deep neural networks. MVCNN [20] project 3D point clouds from different perspectives through spatial projection to obtain 2D images, and then process 2D data based on traditional convolutional neural networks. ShapeNets [21], VoxNet [22], and Vote3deep [23] voxelized the unordered point clouds, and then perform feature learning through the 3D Convolutional Neural Network. VB-NET [24] converts the original point cloud into voxels, uses the pre-trained Voxnet as the feature extractor to extract features from the voxels, and then applies BLS (Broad Learning System) to classify objects. Although these works have achieved good results, there is a dilemma between 2D convolution and 3D convolution: 2D convolution fails to capture 3D geometry information such as normal and shape while 3D convolution requires heavy computation. BCVS [25] uses K-means clustering to divide non-planar patches into planar sub-patches.The largest one among the planar sub-patches replaces the normal and barycenter properties of the voxel with those of itself.

### 2.2. Consuming Point Clouds Directly

In view of the above reasons, a series of recent works [26,27,28,29] have proposed the end-to-end deep learning architecture that effectively processes point clouds. PointNet [9] is a pioneer in processing directly with unordered point sets, the core idea of which is to extract the feature of a single point cloud through vanilla multilayer perceptron(MLP) layer and to integrate the point clouds in the high layer to simulate the symmetric function to form the global feature. However, it treats each point separately and essentially discards the local geometric information that is critical to the effect of the convolutional structure.

In order to solve this problem, some new methods are proposed quickly. As an extended version of PointNet [9], PointNet++ [10] proposes a hierarchical neural network structure based on ball query and FPS (farthest point sampling), which optimizes the learning performance of local features of the network. LKPO-GNN [30] transforms the disordered three-dimensional point cloud into an ordered one-dimensional sequence, reducing the complexity of calculation. PointfusionNet [31] fuses point features with corresponding local features, and maps them to a higher-dimensional space to extract more rich local features. ShufflePointnet [32] can exploit fine-grained local features while using group convolution and channel shuffle operation to reduce redundancies. TGNet [33] performs local convolution on irregular point sets and improves scale invariance by extracting fine-grained semantic features from multi-scale neighborhoods.

Despite achieving leading performance in various tasks such as classification and segmentation, the implementation process of these methods is relatively complicated and the design ideas have abandoned some classical deep learning networks, like GoogleNet [34], ResNet [35], etc. These classical networks all have mature network structure and learning performance, which play an important role in promoting the development of deep learning. Therefore, we suspect that the classical network approach can be easily applied to point cloud processing tasks with some improvements. In this paper, we directly take raw point sets as input to avoid the combinatorial irregularities and complexities of meshes. We use the novel MSA (modified set abstraction) module as the feature extraction backbone of the model, which uses similar methods in [10] but with some improvements described in Section 3.

## 3. Methods

This paper introduces a novel and general framework for 3D point cloud processing that includes 3D shape classification and scene semantic parsing. The proposed framework directly takes raw point sets as input. Point sets are the collection of multiple points, each of which contains the $\left(x,y,z\right)$ coordinate information.

Combined with the recent development in deep learning on point clouds and the idea of classical networks, the method can better capture local feature information and improves the model performance by further learning between neighboring points and further integration between high and low levels. The first section describes how the feature extraction layer constitutes the MSA module. The second section explains the specific design of the R3Block Layer. The last section proposes the overall architecture of the method.

### 3.1. MSA Module

**Figure 1:**
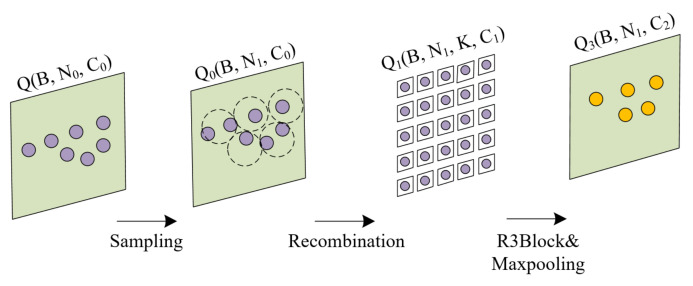
The MSA module structure. From left to right, the processing flow charts of Sampling layer, Recombination layer, R3Block layer, and Maxpooling layer are in order.

In this section, we present our MSA module modified by set abstraction for point cloud processing. The feature extraction layers are composed by a series of MSA modules. As shown in Figure 1, each MSA module is made of four layers: Sampling layer, Recombination layer, R3Block layer, and Maxpooling layer. The sampling layer selects a fixed number of point clouds which define the centroids of the local area. Next, the Recombination layer is used to find neighboring points around the centroids. The R3Block layer adopts the residual-like structure to encode local features and fuse the information of different feature layers. Finally, the maxpooling layer achieves feature dimensionality reduction and expression of each centroid point.

We assume that the input point clouds of each MSA module $\left\{x_{1},x_{2},\ldots ,x_{n}\right\}$ are expressed as *Q*, which is a matrix of $B\times N_{0}\times C_{0}$. *B* is the batch size, $N_{0}$ represents the number of point clouds in each sample, and $C_{0}$ represents the number of channels. First, the sampling layer choses $N_{1}$ point clouds as centroids of the input point clouds by the FPS. The output of the sampling layer is $Q_{01}$, and its size is $B\times N_{1}\times C_{0}$. Then, the recombination layer selects *K* neighboring points for each centroid by Ball Query, which finds all points that are within a radius to the query point, and fuses the coordinate information of each neighboring point with the information of the $C_{0}$ feature channels. The recombination layer generates a new output $Q_{11}$, which is a matrix of $B\times N_{1}\times K\times C_{1}$. In addition, the R3Block layer further extracts features from point set. In the whole feature extraction process, the number of centroids and neighboring points remains the same, the only change is the corresponding feature dimension $C_{1}$ of each point. The output size of the R3Block layer $Q_{2}$ is $B\times N_{1}\times K\times C_{2}$. Finally, we employ the maxpooling layer as the symmetric reduction function to aggregate information about neighboring point clouds. The output of the MSA module is $Q_{3}$, which is a matrix of $B\times N_{1}\times C_{2}$. In this case, the symmetric reduction function can be defined in Equation (1):

(1) $$Q_{3i}\overset{\underset{}{}}{=}\left\{max\left(Q_{2ij}\right)|i=1,...,N_{1};j=1,...,k\right\}$$

Although the structure of the MSA module we designed is a bit similar to the set abstraction module of PointNet++, it is actually different. This is mainly reflected in the R3Block layer for encoding local features, which is based on the idea of the classical residual block. We will introduce the more design details of the R3Block layer in the following section.

### 3.2. R3Block Layer

In order to better capture the local structure and improve network performance, we hope to further learn the geometric information of the point sets. PointNet++ [10] introduces the hierarchical neural network structure, which recursively applies the Mini-PointNet to the nested partitioning of the input point sets, so that the model can better learn the local features by increasing the contextual scales. It still draws on the basic idea of PointNet: the model first learns the spatial encoding of each individual points based on the MLP strategy, and then uses the pooling layer to aggregate the neighborhood information to realize the perception of local structure. However, this idea is not fully considered in terms of capturing the local features. More specifically, PointNet++ aggregates the features of the neighboring points after acquiring the neighboring points for each centroid to effectively enhance the ability of local feature learning, but the learning of neighboring points are independent of each other for each centroid point. This will lead to the problem that the system is not robust enough when the number of samples is reduced. Therefore, we draw on the idea of the classical network to allow neighboring points of each centroid to learn from each other to further capture the local structural information while improving the selectivity and adaptability of the network learning process.

In the design of GoogleNet Inception v3 [36], the network decomposes the n×n convolution kernel into 1×n and n×1, achieving the same performance while reducing the number of parameters. Referring to the ideal and extensive experiments, we use the 1×3 convolution kernel to learn from neighboring clouds, and then use the 1×3 convolution kernel to further learn local features. In order to ensure that the feature dimensions between the network layers remain the same, the padding of the two convolutional layers with the 1×3 convolution kernel is “SAME” and the stride is set to 1.

**Figure 2:**
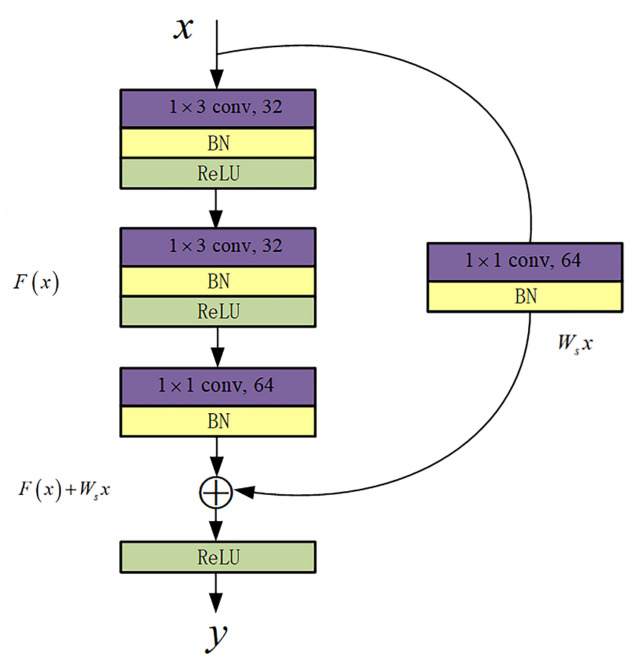
The R3block layer structure. The layer draws on the idea of residual block, which is used to fuse the features of neighboring points around the centroids better.

The operation of increasing the dimension is then implemented using the convolutional layer with the 1×1 convolution kernel, where the padding is “VALID” and the stride is set to 1. The 1×1 convolution kernel not only reduces the number of parameters, but also the key for neighboring points to learn from each other. It can increase the feature channels, which is equivalent to combining the features of independent neighboring points, so that neighboring points are not independent of each other anymore. This small detail can make the network better extract local features and have better robustness when the number of sampled point clouds is reduced. The network’s expression ability is also improved by using the nonlinear activation function to increase the nonlinearity while maintaining the size of the feature map. Finally, we draw on the ideal of the bottleneck architectures of ResNet [35]: a convolutional layer with the 1×1 convolution kernel is connected in parallel between the input and output of the module. The padding is “VALID” and the stride is 1. On the one hand, the adoption of this method of projection solves the problem of dimensional mismatch. On the other hand, by fitting the residual function, the problem of network degradation is prevented while improving the selectivity and adaptability in the network learning process. The specific structure is shown in Figure 2 as an example.

We explicitly make these layers fit the residual mapping, rather than expecting each of few stacked layers to fit into the desired underlying mapping directly. As shown in Figure 2, we consider G(x) as the underlying mapping to be fit through several stacked layers, where *x* is the input of the R3block layer. The dimensions of *x* and *F* are not equal, while the channel between the input and output changes. We use a shortcut connection $W_{s}$ for linear projection to match the dimensions. These stacked nonlinear layers is used to explicitly approximate the residual functions $F\left(x\right)=G\left(x\right)-W_{s}x$. Then the original mapping becomes $F\left(x\right)+W_{s}x$. The operation of $F\left(x\right)+W_{s}x$ is done by adding elements together, followed by the nonlinear activation function. Although both forms should be able to approximate the required function asymptotically, the ease of learning may be different. The definition of the R3block layer is as shown in Equation (2):

(2) $$y\overset{\underset{}{}}{=}\delta \left(F\left(x,\left\{W_{i}\right\}\right)+W_{s}x\right)$$

### 3.3. Overall Architecture

The approach we proposed directly consumes the disordered point sets $\left\{p_{1},p_{2},\ldots ,p_{n}\right\}$, where each point $p_{i}$ has three feature channels that are coordinate information of the x, y, and z axes, respectively. The network can be divided into the front-end part and the back-end part. The front-end part is the main body of the network for feature extraction. The main body of the network includes some MSA modules, and the modules are hierarchically nested. Each MSA module is composed of the Sampling layer, the Recombination layer, the R3Block layer, and the Maxpooling layer. The centroid points in each MSA module is selected from the output of the previous MSA module by FPS, and the number is gradually reduced. As the selection radius of the ball query gradually increases, the feature dimension output by each module also gradually increases.

**Figure 3:**
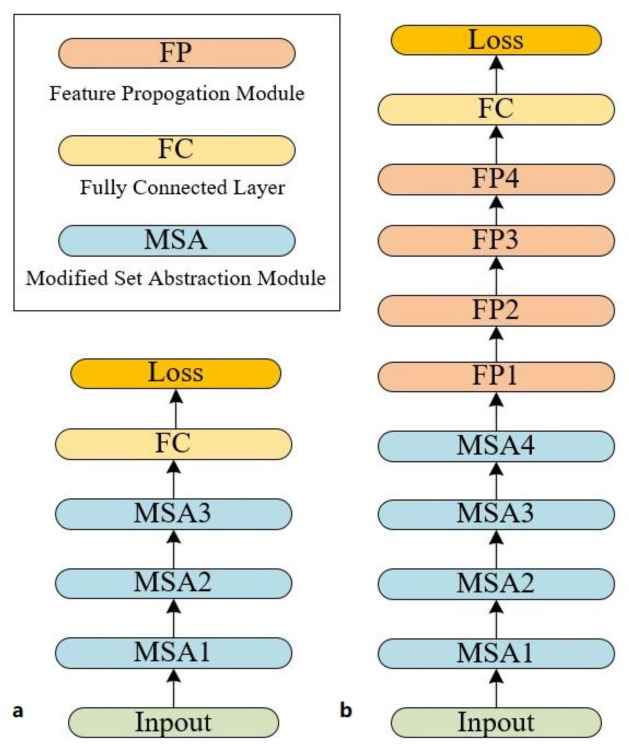
Proposed network architecture for classification (**a**) and segmentation (**b**) tasks. Where the part stacked by the MSA module is the front-end of the network model, and the other part is the back-end.

As shown in Figure 3, there are green connections on the left side of the front-end part of the network, which represent the index layers of the point clouds from the upper layers. The index layers are used for the selection of the centroid points and neighboring points. Such an operation causes neighboring points to be mapped to a higher dimensional space, while the representation of the point clouds is redundant, but it can prevent the loss of information during maxpooling operations. Adequate information about point clouds is preserved during feature extraction, which facilitates the network to further digest the information to obtain the characteristics of point sets.

As shown in Figure 3, the back-end part is the functional module of the network, which completes the classification and segmentation tasks, respectively. In the classification task, the back-end part consists of three fully connected layers, and the final result is the category to which the predicted object belongs. C represents the number of different object classes in Figure 3a. S indicates the type of object to which the input object belongs and element $S_{j}$ represents probability that the input object belong to object of class *j*. In the face of different tasks, the way of feature fusion and prediction processing at higher levels is naturally different. In the segmentation task, the back-end part consists of four FP modules (Feature Propagation Modules) and two 1-d convolution layers.

The final result is the category to which each point clouds belongs. The FP structure is applied in PointNet++ [10]. The structure is similar to U-NET [37], and the underlying features are fused with high-level deconvolution features to obtain the better segmentation effect. In Figure 3b, M represents the number of categories of labels of the point clouds in the scene. F indicates the label to which each point belongs and element $F_{ij}$ represents probability that *i* the point belongs to object of class *j*. Moreover, for deep networks, the most critical point is to produce high-dimensional sparse representations, which means that these deeper layers require local processing. The 1×1 convolution kernel can achieve information fusion between channels without increasing the number of parameters while obtaining good sparsity. Therefore, we did not replace the original FP structure based on the 1×1 convolution kernel with R3Block layer in the higher-dimensional convolutional layer.

In the proposed approach, we use the cross-entropy as the classification loss function as shown in Equation (3):

(3) $$L=-\sum_{n=1}^{N}y_{c}log\left(p_{c}\right)$$

*N* represents the number of object categories, and $y_{c}$ is a one-hot vector representing *N* categories. If the category is the same as the sample category, then take 1; otherwise, take 0. $p_{c}$ represents the probability that the predicted sample belongs to *c*.

## 4. Experiment Result and Discussion

In the experimental section, the experimental results of our framework on a number of benchmark datasets would be presented. In Section 4.2, we performed extensive evaluation of our model for classification tasks on the ModelNet40 [21] datasets. In Section 4.3, experiments are extended to the scene semantic segmentation task on the ScanNet [38] datasets. In Section 4.4, we evaluate the robustness of our approach under incomplete sampling by reducing the number of input points. All of our experiments are conducted on an Intel Xeon Silver 4110 2.1GHz CPU with one Tesla T4 GPU.

### 4.1. Datasets

Before we dive into the experimental results, we will introduce the datasets:

- 3D Object datasets: ModelNet40 is the benchmark dataset for 3D shape classification tasks, which is composed of 12,311 CAD models from 40 man-made object categories. We use the point cloud conversion of ModelNet40 provided by [9], where the 1024 uniformly sampled points from the mesh surface of each model according to face area are normalized into a unit sphere. We use the official split with 9843 shapes for training and 2468 for testing.
- Indoor remote-sensing datasets: ScanNet is a semantic scene labeling task with a total of 1513 scanned scenes. There are a total of 21 category labels for point sets in the dataset. Each point of the dataset is annotated with one of the semantic labels without RGB information. We follow the experiment setting in [10] and take 1201 scenes for training and 312 scenes for testing.

### 4.2. Classification Results

We evaluated proposed network on the ModelNet40 dataset to evaluate the model performance for object classification. Specific parameter settings: the optimization method is set to Adam optimizer with batch size of 16 and initial learning rate of 0.001. The momentum is set to 0.9, and the learning rate decays every 200,000 steps by a factor of 0.7. For the classification model, the number of MSA modules is set to 3.

Table 1 compares the accuracy between proposed method and existing methods. The column “input” represents the format of the input, “Accuracy avg. class” and “Accuracy overall” indicate the average accuracy and overall accuracy in Table 1, respectively. We use the (x, y, z)-coordinates of the 1024 points as the input for the experiments. These methods use different representations of object data and perform different core operations. The method we proposed based on classical network ideas outperforms most existing methods; specifically, the overall accuracy of the proposed method is 8.1% and 6.9% higher than ShapeNets and VoxNet, respectively. Compared with point clouds, mesh needs to choose the type of facets and how to connect, and volume needs to choose its own size and resolution, which requires a lot of computation, and causes some spatial information loss and limits their accuracy.

Although PointNet and PointNet++ deal with raw point sets directly, they abandon the ideas of classical networks. The average accuracy of our method is 1.9% higher than that of PointNet++, while the overall accuracy is 2.1% higher than that of PointNet++. Since the processing process is more delicate and the feature extraction is sufficient, our method even outperforms SpiderCNN [39] by 0.4% in overall accuracy.

### 4.3. Segmentation Results

To further validate the effectiveness and generalization ability of our model, we apply the model trained on the indoor remote-sensing dataset named ScanNet for the segmentation task. We take each scene as the unit sample, and randomly sample 8192 point clouds in each sample. The number of MSA modules is set to 4. Other specific parameter settings are consistent with the above experiment. Table 2 shows the segmentation results of proposed method compared with other methods. We report mean intersection over union (IoU) and overall accuracy (Accuracy); 3DCNN has high space and time complexity at high resolutions and more quantization errors at low resolutions, which greatly limits its network performance. In this work, we apply the residual-like block to fuse the feature information of neighboring points while dealing with the unordered point sets directly. As can be seen from the Table 2, our method improved by 1.1% in accuracy and 3.24% in mean IoU. The experimental results show that the network model performs well in the task of semantic segmentation.

**Figure 4:**
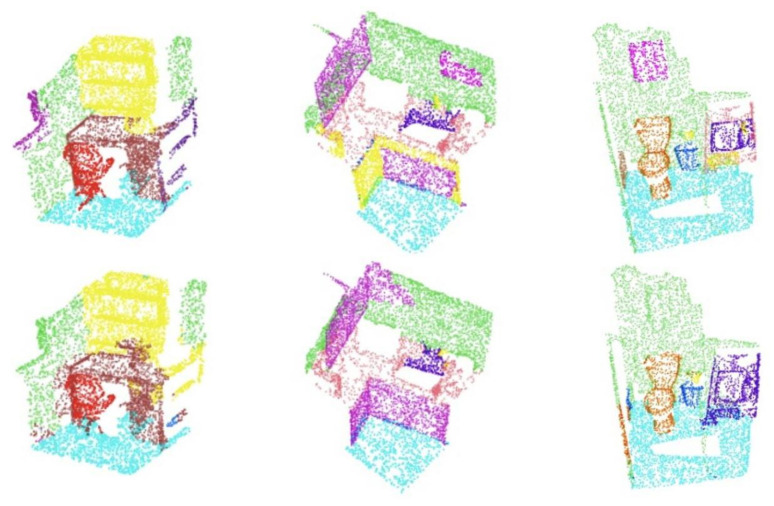
Randomly sampled point cloud from the ModelNet40 dataset. The pictures in the top row are the ground truth, and the pictures in the bottom row are the prediction. Different colors represent different object labels.

We randomly select some scenes in the test set of the indoor remote-sensing datasets and visualize the results of these scene segmentation using our model. As shown in the Figure 4, our model performs semantic segmentation tasks on the indoor scene, with the original renderings in the upper row and the predicted renderings in the lower row. Although there are a few errors in the prediction of certain points, our segmentation results are still satisfactory compared with the ground truth in these indoor scenes.

### 4.4. Robustness Testing

**Figure 5:**
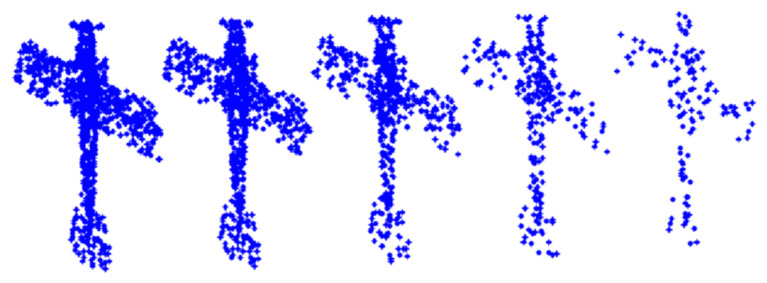
Semantic segmentation results of some scenes in the ScanNet dataset. From left to right, the number of samples of the point clouds is 1024, 768, 512, 256, 128.

**Figure 6:**
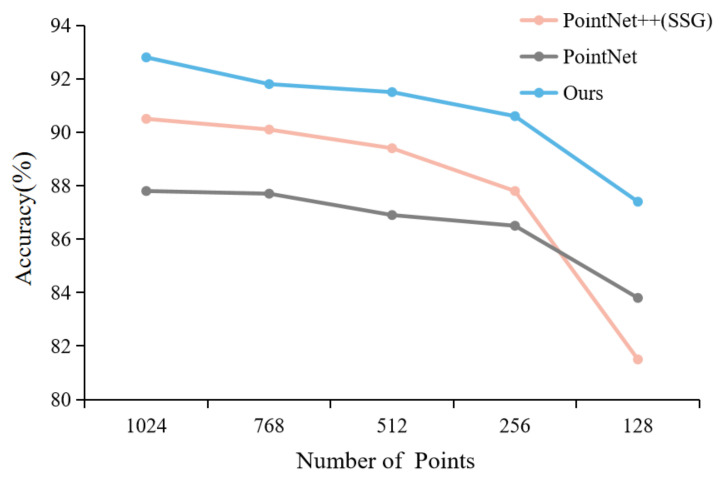
Classification accuracy of our model and other models with different numbers of input points on ModelNet40 dataset.

Data captured directly by sensors from real world often encounters severe incomplete sampling problems. In order to verify the robustness of proposed network to non-uniform and sparse data, we evaluated the performance of the proposed model under different input points in the ModelNet40 classification task by comparing with PointNet and PointNet ++ (SSG). Specifically, we choose the point cloud model in the ModelNet40 dataset. Through uniform sampling of point cloud data and point cloud data of 1024, 768, 512, 256, 128, five cases were collected. As shown in Figure 5, taking the data of the aircraft model as an example, the number of samples from left to right is gradually reduced. As shown in Figure 6, the experimental performance of our model at 1024 input points with 92.8% accuracy is comparable to that of PointNet ++. In addition, when the number of input points is further reduced to 128, our model with 87.4% accuracy outperforms PointNet by 3.6%, which is better than PointNet ++ 5.9%. Through robust experiments with reduced sampling points in a single dataset and comparative experiments with other methods in different datasets, we demonstrate that the proposed method can efficiently and robustly learn local geometric features, which improves the robustness of the random point dropout.

## 5. Conclusions

In this paper, a novel and general network model was proposed, which is applied to the point cloud processing by improving the classical network. Since point clouds are unordered and irregular, classical networks cannot be used to handle them directly. We constructed the MSA module and used a R3Block layer to replace the MLP-based mini PointNet layer to encode the local feature vectors, implicitly integrating the features among points in the local region. Through robust experiments with reduced sampling points in a single dataset and comparative experiments with other methods in different datasets, we demonstrate that the proposed method can efficiently and robustly process raw point sets on both 3D object datasets and indoor remote-sensing datasets. Specifically, the prediction accuracy of proposed method in the ScanNets and ModelNet40 datasets can reach 84.5% and 92.8% respectively. When the number of sampled point clouds is reduced from 1024 to 128, the prediction accuracy of proposed method can still maintain 87.4%. The experimental results demonstrate that the performance of proposed network is not only better than other point cloud based methods on the semantic segmentation task, but also has better robustness when the number of input point clouds is reduced. Through feature extraction between point clouds in the local domain, global features and local features can be effectively fused. The network model proposed in the point sets processing tasks has achieved on par or better performance than most existing networks.

However, the method proposed in this research still has many shortcomings, such as limitations in scene feature extraction. This method essentially only considers the correlation between point clouds, and does not fully reflect the original geometric structure of point clouds. At the same time, the FPS method in the paper now has more optimization methods that can be improved, which will be our future exploration direction. Facts have proven that only by fully restoring the original geometric structure of the point cloud can the characteristics of the point cloud be fully expressed. We will do more exploration in this area in the future.

## Figures and Tables

**Table 1:** Comparison of Classifification results on ModelNet40.

Method	Input	Accuracy Avg. Class	Accuracy Overall
ShapeNets [21]	Volume	77.3	84.7
VoxNet [22]	Volume	83.0	85.9
VB-Net [24]	Volume	84.0	-
LFD [21]	Image	75.5	-
MVCNN [20]	Image	-	90.1
PointNet [9]	Point	86.2	89.2
PointNet++(ssg) [10]	Point	87.6	90.7
SpiderCNN [39]	Point	-	92.4
LKPO-GNN [30]	Point	88.2	90.9
Ours	Point	89.5	92.8

**Table 2:** Comparison of Segmentation results on ScanNet dataset.

Method	Input	Accuracy Overall	Mean IoU
3DCNN [38]	Volume	73.0	13.5
OctNet [40]	Volume	76.6	26.4
PointNet [9]	Point	73.9	17.9
TCDP [41]	Point	80.9	-
PointNet++(ssg) [10]	Point	83.4	38.28
RSNet [42]	Point	-	39.35
ShellNet [43]	Point	85.2	-
Ours	Point	84.5	41.52

## Data Availability

Not applicable.
